# Characterisation of *Lagovirus europaeus* GI–RHDVs (Rabbit Haemorrhagic Disease Viruses) in Terms of Their Pathogenicity and Immunogenicity

**DOI:** 10.3390/ijms25105342

**Published:** 2024-05-14

**Authors:** Beata Tokarz-Deptuła, Jakub Kulus, Łukasz Baraniecki, Michał Stosik, Wiesław Deptuła

**Affiliations:** 1 Institute of Biology, University of Szczecin, 71-412 Szczecin, Poland; lukaszjozefbaraniecki@gmail.com; 2 Institute of Veterinary Medicine, Faculty of Biological and Veterinary Sciences, Nicolaus Copernicus University in Torun, 87-100 Torun, Poland; jakub.kulus@umk.pl (J.K.); wieslawdeptula@umk.pl (W.D.); 3 Institute of Biological Sciences, Faculty of Biological Sciences, University of Zielona Gora, 65-516 Zielona Gora, Poland; m.stosik@outlook.com

**Keywords:** rabbit, RHDV, immunology, phylogenogroups, immunotypes, haematotypes, pathotypes

## Abstract

Rabbit haemorrhagic disease viruses (RHDV) belong to the family *Caliciviridae,* genus *Lagovirus europaeus*, genogroup GI, comprising four genotypes GI.1–GI.4, of which the genotypes GI.1 and GI.2 are pathogenic RHD viruses, while the genotypes GI.3 and GI.4 are non-pathogenic RCV (*Rabbit calicivirus*) viruses. Among the pathogenic genotypes GI.1 and GI.2 of RHD viruses, an antigenic variant of RHDV, named RHDVa—now GI.1a–RHDVa, was distinguished in 1996; and in 2010, a variant of RHDV—named RHDVb, later RHDV2 and now GI.2–RHDV2/b—was described; and recombinants of these viruses were registered. Pathogenic viruses of the genotype GI.1 were the cause of a disease described in 1984 in China in domestic (*Oryctolagus* (*O.*) *cuniculus domesticus*) and wild (*O. cuniculus*) rabbits, characterised by a very rapid course and a mortality rate of 90–100%, which spread in countries all over the world and which has been defined since 1989 as rabbit haemorrhagic disease. It is now accepted that GI.1–RHDV, including GI.1a–RHDVa, cause the predetermined primary haemorrhagic disease in domestic and wild rabbits, while GI.2–RHDV2/b cause it not only in rabbits, including domestic rabbits’ young up to 4 weeks and rabbits immunised with rabbit haemorrhagic disease vaccine, but also in five various species of wild rabbits and seven different species of hares, as well as wild ruminants: mountain muskoxen and *European badger*. Among these viruses, haemagglutination-positive, doubtful and harmful viruses have been recorded and described and have been shown to form phylogenogroups, immunotypes, haematotypes and pathotypes, which, together with traits that alter and expand their infectious spectrum (rabbit, hare, wild ruminant, badger and various rabbit and hare species), are the determinants of their pathogenicity (infectivity) and immunogenicity and thus shape their virulence. These relationships are the aim of our consideration in this article.

## 1. *Lagovirus europaeus* GI–RHDV Characteristics

**Figure 1:**
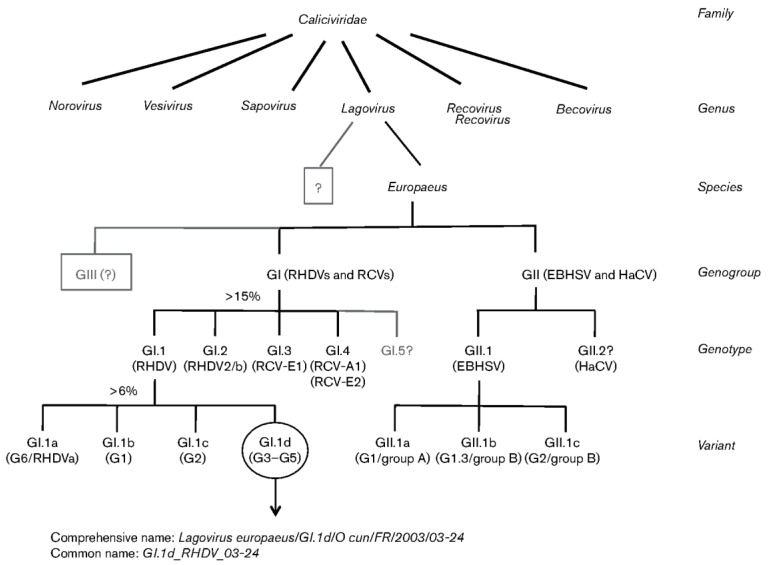
Proposed taxa organisation of the Lagovirus genus within the Caliciviridae family [1]. Recognised entities are in black print, whilst potential new groups, yet to be discovered, are indicated in light grey with a question mark. The GII.2 genotype is denoted with a question mark since it is still based on a single available VP60 sequence. *Lagovirus europaeus* constitutes the single species of known lagoviruses. It contains two genogroups that can be subdivided into genotypes, further subdivided into variants. Genogroups are denoted with roman numerals, genotypes with Arabic numerals and variants with letters. The minimal genetic distances required to distinguish genotypes and variants are indicated as percentages. Current nomenclature is shown in parentheses. The proposed comprehensive and common names of one strain taken as an example (circle) are indicated. Descriptions: see main text for details.

According to the currently proposed systematics (Figure 1), within *Lagovirus europaeus* GI–RHDV viruses, which belong to the family Caliciviridae, a distinction is made between the genogroup GI–RHDV/RCV (rabbit haemorrhagic disease virus/rabbit calicivirus) and the genogroup GII–EBHSV/HaCV (European brown hare syndrome virus/hare calicivirus), in which genotypes have been distinguished. In the genogroup GI–RHDV/RCV, four genotypes have been described (GI.1–GI.4), comprising the genotype GI.1, including the antigenic variant of these viruses GI.1a–RHDVa, which are pathogenic viruses, and the genotype GI.2, which are also pathogenic viruses previously identified as RHDV variants—RHDVb or RHDV2, now GI.2–RHDV2/b. In contrast, the genotypes GI.3 and GI.4 are non-pathogenic RCV viruses, including RCV–E1, RCV–E2 and RCV–A1 [1]. On the other hand, in the genogroup GII–EBHSV/HaCV, two genotypes (GII.1 and GII.2) are distinguished, except that the genotype GII.1 represents EBHS viruses pathogenic to hares, within which three variants are distinguished (GII.1a, GII.1b and GII.1c), while the genotype GII.2 is represented by HaCV viruses non-pathogenic for these animals, which show 79% similarity to viruses of the genotype GII.1–EBHS [1,2]. However, according to Mahar et al. [3], they form, in addition to the genotypes GII.2, GII.3 (HaCV–A1), GII.4 (HaCV–A2) and GII.5 (HaCV–A3) [3].

Characterising the pathogenic *Lagovirus europaeus* viruses of the genogroup GI–RHDV and the genotypes GI.1 and GI.2, it should be noted that they most likely evolved from the apathogenic rabbit intestinal calicivirus [4]. Currently, within the viruses of the genotype GI.1–RHDV, four variants (GI.1a–GI.1d) and six genetic groups (G1–G6) have been distinguished, to which the four variants mentioned above—G6 (GI.1a), G1 (GI.1b), G2 (GI.1c) and G3–G5 (GI.1d)—have been assigned [1,5]. According to French and Italian authors [6], these four variants (GI.1a, GI.1b, GI.1c, GI.1d) of GI.1–RHDV form a single serotype. It has been shown that the most diverse and distinct among these four viral variants of the GI.1–RHDV genotype is the GI.1d variant, which is most closely related to the GI.1a variant [7]. It has also been recorded that these four GI.1a–GI.1d–RHDV variants are differentiated in terms of pathogenicity (infectivity), as the GI.1a variant viruses, previously referred to as antigenic variant RHDVa [8,9], are the causative agents of rabbit haemorrhagic disease in different regions of the world [1,4,8,10,11]. In contrast, GI.1b variant viruses have been the cause of this disease in rabbits from the Iberian peninsula [11], while viruses of the GI.1c variant are identified with the first pathogenic viruses found in rabbits with this disease in China, which have been termed either RHDV or RHDV1 viruses [11,12]. Meanwhile, the viruses forming the GI.1d variant have been the cause of haemorrhagic disease in rabbits in only some European countries [11]. In the case of *Lagovirus europaeus* GI–RHDV of the GI–RHDV/RCV genogroup, genotype GI.2, they are represented by the pathogenic GI.2–RHDV2/b viruses, previously referred to as a variant of RHDV–RHDVb or RHDV2 [13], within which variants and genetic groups have not been distinguished [1]. These viruses show slightly less pathogenicity in rabbits than GI.1–RHDV, including GI.1a–RHDVa, although according to Kerr et al. [4], this characteristic is conditioned by their strain type, while according to Buehler et al. [14], it is also related to the immune status of the infected animals. These viruses, i.e., GI.2–RHDV2/b, are now more commonly recorded in the wild relative to previously described GI.1–RHDV viruses, including GI.1a–RHDVa and non-pathogenic RCV viruses defined as GI.3 (RCV–E1) and GI.4 (RCV–A1, RCV–E2) viruses [1,15,16,17,18], which confer partial cross-resistance in rabbits against viruses of the GI.1b–RHDV variant [19]. The latter mentioned non-pathogenic RCV, currently, GI.3 (RCV–E1) and GI.4 (RCV–A1, RCV–E2) [1,11], although not causing fatal declines in experimentally and naturally infected domestic rabbits [10], cause (but only RCV–A1 viruses) fatal declines in wild rabbits [1,15,16,17,18], mild small intestinal infection in domestic rabbits [20,21] and partial serological immunity against GI.1–RHDV viruses, including GI.1a–RHDVa [4,21,22], as well as GI.1c–RHDV viruses [20,22,23,24,25]. It has been shown [26] that these GI.1c–RHDV viruses, together with GI.1a–RHDVa, are now very commonly recorded in rabbit populations in China. It is also indicated [4] that the RCV–A1 viruses in wild rabbits, which do not induce a serological response and do not immunise rabbits to the GI.2–RHDV2/b viruses that are now commonly found in the environment, are probably the reason for GI.2–RHDV2/b viruses showing pathogenic effects in many domestic rabbits (young, vaccinated, adult) and several wild rabbits. Furthermore, it should be added that among the *Lagovirus europaeus* GI–RHDV, viruses that are weakly pathogenic, non-pathogenic or of unclear pathogenicity have also been described, which include the American (Michigan) Ashington virus (MRCV) [1,21], not classified into any genogroup or genotype within the currently proposed systematics of these viruses [1] and the non-pathogenic Lambay virus described in Lambay Island rabbits [27], also not included in the currently proposed systematics of these viruses [1]. Moreover, within Lagovirus europeaus GI–RHDV, the so-called core-like particles (CLPs) referred to as smooth s-RHDV strains are mentioned [28], which are very similar in structure to GI.1–RHDV viruses but are characterised by a smaller diameter of only 25–29 nm, instead of about 30–40 nm, and do not have the characteristic cavities in the capsid [29,30]. These smooth s-RHDV strains show less pathogenicity than GI.1–RHDV viruses because they induce weak disease symptoms and a significantly lower mortality rate in infected rabbits, although they induce partial antibody-related immunity against these viruses [31].

In characterising the pathogenicity of Lagovirus europeaus GI–RHDV, GI.1–RHDV viruses, including GI.1a–RHDVa, have also been found to cause a condition in domestic (*O. cuniculus* domesticus) and wild (*O. cuniculus*) rabbits [6,10,32,33], defined since 1989 by the OIE (World Organisation for Animal Health) as haemorrhagic disease of rabbits [34,35]. This disease in domestic rabbits, imported from Germany, was first described in 1984 in China and then spread to wild rabbits [4,36]. Currently, the disease, which is identified as GI.1–RHDV, including GI.1–RHDVa and GI.2–RHDV2/b viruses due to their tropism not only to the vascular endothelium but also to hepatocytes and macrophages, as well as their broad infectious spectrum, is referred to as rabbit haemorrhagic disease and hyperacute or acute necrotic hepatitis [6,14,37,38,39]. Therefore, GI.1–RHDV viruses, including GI.1a–RHDVa, are assumed to cause this disease in domestic (*Oryctolagus* (*O.*) *cuniculus* domesticus) and wild (*O. cuniculus*) rabbits, while GI.2–RHDV2/b viruses are the cause of the disease in these rabbits, including domestic rabbits up to 4 weeks of age and immunised against the disease, and five different species of wild rabbits (*O. cuniculus*, *Sylvilagus* (*S.*) *audubonii*, *S. nuttallii*, *S. floridanus*, *Romegolagus diazi*) and seven different species of hares (*Lepus* (*L.*) *timidus*, *L. timidus hibernicus*, *L. europaeus*, *L. capensis* subsp. Mediterraneus, *L. corsicanus*, *L. californicus*, *L. alleni*), living on the European continent, including Italy, but also on the American continent, as well as in wild artiodactyl ruminants—mountain muskrats (*Moschus sifanicus*) [4,6,9,10,13,14,16,18,19,26,40,41,42,43,44,45,46,47,48,49,50,51,52,53,54,55,56,57]. Moreover, the GI.4/GI.2 recombinant of the GI.2–RHDV2/b has been proven to infect the *European badger* (*Meles meles*) [54]. It should be added that Lopes et al. [58] in 2014 described an infection in hares (*L. granatensis*) on a background of GI.1–RHDV. Currently, infections and diseases in rabbits caused by GI.1–RHDV, including GI.1a–RHDVa and GI.2–RHDV2/b, are recorded in many countries on all continents [4,6,10,11,15,18,21,33,41,54,59,60,61,62,63,64,65,66] except Antarctica [61]. The spread of these infections and the disease caused by these viruses in the wild is also related to the fact that in 1995, to biocontrol the numbers of wild rabbits, considered pests of cultivated plants, the Czech V351 strain of RHDV (now GI.1–RHDV) was introduced to Australia on a planned basis, and within a short time “moved” to New Zealand, among others [4,10]. It should be added that the genetic material of the original infectious agent, i.e., RHD virus (now GI.1–RHDV), was also found in the Tasmanian devil (*Sarcophilus harrisii*) [67], the Mediterranean mouse (*Mus spretus*), the scrub mouse (*Apodemus sylvaticus*) [68], the Mediterranean vole (*Microtus duodecimcostatus*) and the buzzard (Crocidura russula) [28]. In addition, antibodies to this virus have been recorded in dogs, red foxes, cats, mice, and other unspecified domestic animal species [33].

Pathogenic GI.1-RHDV viruses, including GI.1a–RHDVa and GI.2–RHDV2/b, as well as non-pathogenic RCV viruses representing the *Lagovirus europaeus* GI–RHDV group [1], are single-stranded RNA viruses, having a structure of membraneless cubic (icosahedral) capsid symmetry with an outer diameter of 32–44 nm and an inner diameter of 28 nm and a particle density of approximately 1.35 g/dm^3^ [30]. Their capsid contains 180 copies and comprises 90 protein dimers of the main capsid protein VP60, arranged in capsomeres with T = 3 symmetry, forming 12 pentamers and 20 arch-shaped hexamers, between which are 32 cup-shaped cavities [30,69]. Their genetic material, 7.4 kb in size, is diverse, polyadenylated and positively polarised (+ssRNA), encoding nine genes and consisting of 7437 nucleotides, constituting two open reading frames (ORFs), a large one (ORF1) and a small one (ORF2) [4,11,30,70,71,72]. The large open reading frame, ORF1 in these viruses, spans Nucleotides 10 to 7044, and its 5′ end encodes a 257 kD protein within which proteolytic processes have yielded seven non-structural viral proteins (p16, p23, helicase, p29, VPg, protease and RdRp) [71,72], while its 3′ end, encodes the major capsid structural protein VP60 (VP1) of these viruses [70,71,72,73]. The VP60 protein is also encoded by a subgenomic 2.2 kb RNA (sgRNA) [10], 2200 nucleotides in size, which, by binding collinearly to the 3′ end of the genome of this virus, essentially affects its replication [11,55,74]. In contrast, the small open reading frame, ORF2 in the viruses in question, involves nucleotides located at Positions 7025 to 7378 and encodes the smaller VP10 (VP2) capsid protein of these viruses [70,71,73]. This protein is involved in the packaging of viral genetic material [75], is responsible for the stability of viral RNA [55], facilitates recombination between the structural and non-structural protein-coding genes of these viruses [4], and, like the VP60 protein, is also encoded by sgRNAs [4,10]. In addition to these two VP60 and VP10 proteins of the characterised viruses, a non-structural protein 6 (NSP6) has been described in these viruses, with some specificity for the rabbit kidney 13 (RK13) cell line commonly used in in vitro studies [76]. It has been shown that the NSP6 protein, through the activation of caspase-3, -8 and -9, due to increased expression of the Bax gene, conditions the synthesis of apoptosis-activating protein and downregulates the expression of the Bcl2 gene, responsible for the synthesis of protein inhibiting this process, leading to increased apoptosis and reduced cell viability in the rabbit RK13 line [76].

By analysing the role of the major capsid protein VP60 of these viruses, it was shown to be responsible for binding to the cells of susceptible animals [77] and to be composed of three primary domains, i.e., the N-terminal arm, the S (shell) domain and the P (protruding) domain dividing into two smaller subdomains, P1 and P2, which are characterised by high variability in different isolates of these viruses [30,55]. In addition, it has been recorded that the occurrence of the C-terminal sequence of the VP60 protein on its outer surface is responsible for the antigenic variability of these viruses [55]. It has also been demonstrated that the recombinant VP60 protein of RHD virus (now GI.1–RHDV), termed VLP (virus-like protein), does not differ morphologically and antigenically from the VP60 protein of this virus found in the environment and is used as a ‘carrier’ for many antigens [78,79], including as a carrier in a vaccine against GI.1a–RHDVa and GI.2–RHDV2/b viruses [14,80]. It has been shown that the high variability within the amino acid conformation of the VP60 protein of *Lagovirus europaeus* GI–RHDV viruses has created the possibility of distinguishing among the pathogenic RHD viruses, the antigenic variant RHDV–RHDVa, now referred to as the GI.1a–RHDVa virus, and the variant RHDV–RHDVb or RHDV2, now referred to as the GI.2–RHDV2/b virus [9,13,56], although possibly also other infectious forms, including their recombinants [4,11,54,72], and which has been the cause of the change in their infectious spectrum [6,9,10,13,14,16,19,26,41,42,43,44,45,46,47,48,49,50,51,52,56,81]. Furthermore, it should be added that among the recombinants of characterised viruses, intergenotypic recombinants of pathogenic viruses have been described, namely GI.1a–GI.2, GI.1b–GI.2 and GI.4–GI.2, [4,11,12,54,72] and genotypic recombinants of non-disease and disease-causing viruses, namely GI.4–GI.1a, as well as recombinants of pathogenic viruses of the genotype GI.1 with non-pathogenic RCVs of the genotypes GI.3 and GI.4, namely GI.d–RCV–E1 and G.1.d–RCV–A1 [11,21,54]. Among the recombinants of these viruses, intergenotypic recombinants have also been shown to be registered, possessing, in addition to their own structural and non-structural gene sequences of pathogenic GI.2–RHDV2/b and GI.1–RHDV, including GI.1b–RHDV, the non-structural genes of non-pathogenic RCV viruses [12,18,21,47,54,72,82,83,84]. Also found among pathogenic recombinants in hares is a recombinant GI.2–RHDV2/b, carrying non-structural gene sequences of the GII.1–EBHSV virus [72].

## 2. Features of *Lagovirus europaeus* GI–RHDV Viruses in Terms of Their Pathogenicity (Infectivity) and Immunogenicity

Among the essential features of pathogenic microorganisms, including viruses, are their pathogenicity (infectivity) and immunogenicity, which determine their virulence, and the knowledge of which not only sheds light on their pathogenicity and the diseases caused by them but also provides opportunities to develop effective countermeasures against them. To clarify what determines and influences the pathogenicity and immunogenicity of *Lagovirus europaeus* GI–RHD viruses, their characteristics were analysed in this context: the ability to haemagglutinate erythrocytes, the formation of phylogenogroups, immunotypes, haematotypes and pathotypes and the change and extension of the infectious spectrum resulting from their formation of the antigenic variant RHDV–RHDVa (the GI.1a–RHDVa) and the RHDV–RHDVb or RHDV2 variant (now GI.2–RHDV2/b).

### 2.1. Haemagglutination Capacity of Lagovirus europaeus GI–RHDV Viruses and Their Pathogenicity (Infectivity) and Immunogenicity

**Figure 2:**
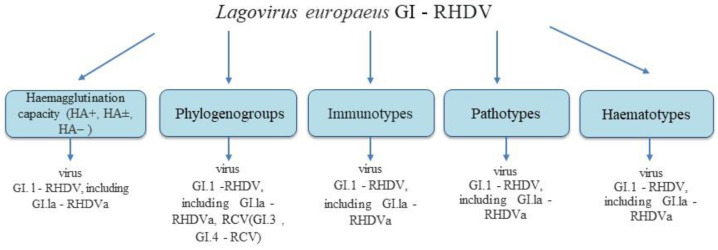
*Lagovirus europaeus* GI-RHDV genotypes and variants affecting pathogenicity and immunogenicity, characterised by haemagglutination ability and forming phylogenogroups, immunotypes, haematotypes and pathotypes.

Analysing the haemagglutination capacity of these viruses (Figure 2), it should be noted that, in addition to the GI.2–RHDV2/b viruses [26] and CLP core particles–s-RHDV strains [29,72], pathogenic GI.1–RHDV viruses, including GI.1a–RHDV, show the ability to bind to erythrocytes of all human blood groups, mainly blood group “0” [85,86,87]. It has been documented that of the more than 500 GI.1–RHDV viruses described, including GI.1a–RHDVa [88], most of them can haemagglutinate erythrocytes, and they are referred to as haemagglutination-positive viruses (HA+) [10,85], and only a few GI.1–RHDVs, including GI.1a–RHDVa viruses, have been shown to lack this property and are termed haemagglutination-negative (HA−). This group of HA− viruses is represented by the English Rainham virus [59], the Polish BlaszkI virus [63], the Spanish Asturias virus [89], the German Frankfurt virus [90], the French 99-05 virus [91], the Chinese henn-1 virus [92] and the Italian Bg97 [91] and Pv97 viruses [9]. An even smaller group of viruses (but only among GI.1–RHDV viruses) showing erythrocyte agglutination under appropriate conditions has been recorded and have been named haemagglutinating-doubtful (HA+/−) viruses, including the German Hagenow virus [90] and the Polish ŻD virus [93]. It should be added that despite not distinguishing among GI.2–RHDV2/b viruses, the HA+, HA− and HA+/− viruses, a haemorrhagic disease in rabbits caused by the GI.2–RHDV2/b virus, defined as HA−, was described in 2020 in China [26].

When discussing the haemagglutination capacity of *Lagovirus europaeus* GI–RHD viruses, it has been shown that this feature of the viruses is related to and results from the structure of their VP60 protein [92,94,95], in particular, the amino acids located at Positions 416, 423 and 476 [95], as well as at Positions 304, 305, 309, 359, 365, 369, 386 and 470 of the P2 subdomain of this protein [92]. It has been shown [15,16] that an amino acid substitution in the P-domain of the VP60 protein of these viruses, affecting the tertiary structure of this protein, alters its conformation and determines the ability of these viruses to haemagglutinate, which, as shown by the studies of [15,26,33,59,85,91,92,94,95,96,97,98,99,100,101,102] is associated with the pathogenicity and immunogenicity of GI.1–RHDV viruses, including GI.1a–RHDVa, and possibly GI.2–RHDV2/b. It has also been confirmed in studies [92], in which it was shown that HA−GI.1–RHDV viruses, i.e., France-99-05 GI.1–RHDV, Spain AST-89 GI.1–RHDV, and HA−GI.1a–RHDVa viruses, i.e., henn-1 GI.1a–RHDVa, show more significant homology in amino acid structure in the P2 subdomain of the VP60 protein, relative to HA+ GI.1–RHDV viruses, including GI.1a–RHDVa [92]. This lower homology in the amino acid sequence of the VP60 protein of HA+ GI.1–RHDV viruses, including GI.1a–RHDVa, relative to the analogous HA− viruses, considered less pathogenic [92], was confirmed by the presence of nine amino acid conformation differences in pathogenic GI.1–RHDV viruses, including GI.1a–RHDVa, relative to non-pathogenic RCV–A1 viruses [21]. In addition, it is indicated that GI.1–RHDV viruses characterised by a high pathogenicity, probably HA+, show a high rate of change in nucleotides formed, most likely due to the low homology in their amino acid sequences [103]. It should be added that, according to a generally accepted principle in clinical science, microorganisms including viruses, characterised by HA+ ability, are more pathogenic than germs including viruses which are HA− [53,85,96,100]. This relationship between haemagglutination capacity and the pathogenicity of GI.1–RHDV and GI.1a–RHDVa viruses was also confirmed in studies in which it was recorded that the lack of haemagglutination capacity (HA−) of henn-1 GI.1–RHDVa, and essentially the absence of this ability in Rainham GI.1–RHDV, resulted in the intensity of the clinical and pathological changes they induced, including mortality in rabbits infected with them, compared to GI.1–RHDV HA+ viruses, being significantly lower [59,92]. This was also confirmed by the study of Pyon et al. [104], who indicated that the pathogenicity and immunogenicity of different RHDV strains increase with their haemagglutination capacity. It is also consistent with observations [97] indicating the formation of more remarkable changes in haematological patterns in rabbits infected with the more pathogenic GI.1–RHDV HA+ viruses, compared to the infection of rabbits with these HA− and HA+/− viruses. These observations were also confirmed in studies in which GI.1–RHDV HA+ viruses, including GI.1a–RHDVa HA+, were recorded to have a more substantial effect on the immune response of rabbits, as measured by phagocytosis, autophagy—mitophagy and apoptosis—as well as T and B lymphocyte counts, compared to the corresponding HA− viruses [98,99,100,105]. Hence, when assessing the haemagglutination capacity of GI.1–RHDV viruses, including GI.1a–RHDVa, in the context of their pathogenicity and immunogenicity, it must be concluded that such a relationship exists, which, with the remarkable ease of its determination in these viruses, and which is confirmed by its determination in the routine diagnosis of rabbit haemorrhagic disease [10,104,106], establishes that it is a method for practical use in the evaluation of the trait being characterised.

### 2.2. Phylogenogroups of Lagovirus europaeus GI–RHDV Viruses and Their Pathogenicity (Infectivity) and Immunogenicity

When discussing the relationship between the occurrence of phylogenogroups, i.e., the phylogenetic differentiation of *Lagovirus europaeus* GI–RHDV viruses, and their pathogenicity and immunogenicity, it should be noted that these features are related to each other, but only in viruses GI.1–RHDV, including GI.1a–RHDVa and RCV (GI.3 and GI.4–RCV) (Figure 2), because such studies are lacking in the case of the GI.2–RHDV2/b virus. It is due to and related to the nucleotide structure of the individual fragments of the genetic material, amino acid sequences of the proteins of these viruses, or both, resulting in the distinction among them of phylogenogroups and lineages and clades that differ in pathogenicity. This feature determines the immunogenicity of the viruses, as illustrated by the immune response of infected rabbits [8,16,60,102,107,108]. Studies on the occurrence of phylogenogroups among Lagovirus europaeus GI.1–RHDV viruses show that the number of phylogenogroups found within the viruses studied is related to the type of genetic material fragment on which the analysis is based, the number of viruses analysed and their geographical origin and time of occurrence in the environment [5,16,60,65,108,109,110,111,112]. It is confirmed by the analysis of the results of studies [110] concerning the comparison of nucleotide sequences at Positions 6412–6809 of 44 RHDV viruses (now GI.1–RHDV) unspecified in terms of haemagglutination (HA) of erythrocytes, recorded between 1987 and 1995 in Europe, Asia and America, which showed the presence of three phylogenogroups among them. In contrast, the evaluation, concerning the comparison of nucleotide sequences at Positions 6135–6719 of 58 GI.1–RHDV viruses, also indeterminate in terms of HA, and of 3 RCV viruses (now GI.3 and GI.4–RCV), which were from Europe, Asia, America and New Zealand from 1955 to 2003, and of 57 GI.1–RHDV viruses, indeterminate for HA, and 2 RCV (now GI.3 and GI.4–RCV viruses), which originated from Europe (mainly UK), America and New Zealand from 1955 to 2001, distinguished eight phylogenogroups [27,111]. Meanwhile, the analysis [5] comparing the nucleotide sequence located at Sites 6473–7011 of 142 GI.1–RHDV viruses with indeterminate HA ability, recorded between 1987 and 2000 in Europe and North America, identified six phylogenogroups. Analogous results [109], as in these recent studies [5], were obtained from the analysis of the entire VP60 protein coding sequence (Nucleotides 5290–7273) of 44 viruses with no specific HA ability from Asia (mainly China) and Europe and America dating from 1984 to 2010, which also yielded six phylogenogroups, within which four Chinese subgroups were additionally distinguished. In contrast, in a study by McIntosh et al. [112], where the entire amino acid sequence of the VP60 protein was compared, 45 GI.1–RHDV viruses, also unrecognised for HA ability from Europe, Asia, New Zealand and America from 1984 to 2005, only two phylogenogroups were distinguished. Meanwhile, the evaluation by Działo et al. [16], analysing a multi-author study based mainly on sequences encoding the VP60 protein, which consisted of about 1500 pathogenic RHDVs (now GI.1–RHDVs), not specified in terms of HA ability, and about 550 non-pathogenic RCVs (now GI.3 and GI.4–RCVs) recorded in Europe, Asia, America, Australia and New Zealand between 1955 and 2013, showed between two and eight phylogenogroups among them. Also, phylogenetic studies [108], which were based on a partial analysis of the amino acid sequence of the VP60 protein located at Sites 340–440 of the hypervariable E region of 52 GI.1–RHDV viruses, also indeterminate in terms of HA capacity, and of 2 RCV viruses (now GI.3 and GI.4–RCV) from different continents of the world from 1984/85 to 2013, showed four phylogenogroups among them, depending on the place of origin and time of receipt. The authors [108] report that Phylogroup one is formed only by RCV viruses from the UK, Phylogroup two by RHD viruses (now GI.1–RHDV) from different continents, having similarities with the first described RHD viruses and the 1996 RHD viruses from Saudi Arabia. In contrast, Phylogenogroup three is formed by viruses from Saudi Arabia obtained between 2012 and 2013 and RHD viruses from Europe from the last decade of the 20th century, while Phylogenogroup four is formed by RHD viruses described in China as the primary aetiological agent of rabbit haemorrhagic disease and viruses from Egypt, France and the Czech Republic. The authors of these studies [108] also showed that the four phylogenogroups distinguished are conditioned not only by the place and time of the establishment of the viruses studied but also by their pathogenicity and immunogenicity, but they were assessed only by their antigenicity. These observations [108] have been confirmed in studies of 7 GI.1–RHDV viruses [65], but with different HA ability, (3 GI.1–RHDV HA+ viruses, 2 HA− viruses, 2 HA+/− viruses) and 11 GI.1a–RHDVa HA+ viruses analysed on the basis of their whole genome, obtained from Poland between 1988 and 2015, which distinguished four phylogenogroups among the 18 viruses studied. They showed that the less pathogenic GI.1–RHDV viruses, relative to GI.1a–RHDVa viruses, represent three phylogenogroups, while the fourth phylogenogroup is formed by GI.1a–RHDVa viruses, which would confirm the data that GI.1a–RHDVa viruses form a separate fourth phylogenogroup [1,106,113] GI.1a–RHDVa viruses have a different structure and pathogenicity from GI.1a–RHDVa viruses, which determines their immunogenicity [8,9,16,53,96,100,107]. Also, the analysis [60] based on the evaluation of the nucleotide sequences encoding the VP60 protein concerning the occurrence of phylogenogroups within 43 GI.1–RHDV viruses with different HA abilities (42 HA+ viruses, 1 HA+/− virus) and 36 GI.1a–RHDVa viruses, also with different HA abilities (15 HA+ viruses, 3 HA− viruses, 18 HA viruses unspecified), and 2 RCV viruses, now GI.3, GI.4–RCV, from different countries in Europe, Asia, America and Australia from 1985 to 2010 showed that these 81 analysed viruses form four phylogenogroups, which do not include the 2 analysed RCV viruses (now GI.3, GI.4–RCV). It is noted that Phylogenogroup one is formed by 4 GI.1–RHDV HA+ viruses, Phylogenogroup two by 21 GI.1–RHDV HA+ viruses, Phylogenogroup three by 17 GI.1–RHDV viruses, including 16 HA+ and 1 HA+/−, and Phylogroup four by 37 viruses, including 1 GI.1–RHDV HA+ and 36 GI.1a–RHDVa viruses (15 HA+, 3 HA−, 18 HA−undefined viruses). This evaluation [60] indicates that the less pathogenic GI.1a–RHDV viruses form the first three phylogenogroups, while the more pathogenic GI.1a–RHDVa viruses previously referred to as RHDV–RHDVa antigenic variants form the fourth phylogenogroup, indicating and confirming the existence of a relationship between the phylogroup formed and the pathogenicity and thus immunogenicity of the viruses studied [8,9,10,11,12,33,107], as it has been shown [60] that GI.1–RHDV viruses form different phylogenogroups concerning GI.1a–RHDVa viruses, characterised by different pathogenicity and immunogenicity. This is also confirmed by observations [4,16,33,53,60,96,100,114] in which it was shown that the less pathogenic GI.1–RHDV, compared to the more pathogenic GI.1a–RHDVa, are characterised by lower immunogenicity, i.e., their ability to induce an immune response both in terms of natural and acquired immunity.

### 2.3. Immunotypes of Lagovirus europaeus GI–RHDV Viruses and Their Pathogenicity (Infectivity) and Immunogenicity

When analysing the relationship between the occurrence of immunotypes, i.e., immunological diversity among *Lagovirus europaeus* GI–RDHV viruses, and their pathogenicity and immunogenicity, it should be noted that such a relationship has been demonstrated only among viruses GI.1–RHDV, including viruses GI.1a–RHDVa (Figure 2) (no such studies have been conducted for GI.2–RHDV2/b and RCV (GI.3, GI.4)), among which immunotypes have been shown, conditioned by the ability to haemagglutinate and the formation of antigenic variants—features that are related to their pathogenicity and immunogenicity ([15,33,60,91,96,115,116,117] and Section 2.1 and Section 2.6). By evaluating the results of studies of 10 GI.1–RHDV viruses [33], including 8 HA+ viruses, 1 HA− virus and 1 HA+/− virus, in terms of 10 parameters of non-specific cellular immunity, 3 of non-specific humoral immunity, 4 of specific cellular immunity and 3 of specific humoral immunity, between 0 and 60 h after infection of the rabbits with these viruses, their immunological diversity was shown, manifested both by an increase, which was recorded twice as often, and by a decrease in the values of the assessed indicators, which fell between 4 and 8 and 56 and 60 h after infection. These changes were the greatest in both the quantity and quality of the parameters studied in rabbits infected with HA+ viruses, successively in parameters in non-specific cellular immunity, specific cellular immunity, non-specific humoral immunity and specific humoral immunity [33]. Based on these changes, three immunotypes were distinguished among the 10 viruses evaluated: I is the most immunogenic, represented by 1 GI.1–RHDV HA+ virus; II is moderately immunogenic, represented by 5 GI.1–RHDV HA+ viruses and 1 HA− virus; and III is the least immunogenic represented by 2 GI.1–RHDV HA+ viruses and 1 HA+/− virus, indicating that the immunogenicity of the viruses studied, and the formation of immunotypes by them, is largely related to their ability to haemagglutinate, a feature that determines pathogenicity (Section 2.1). Also, studies [96] on the immunological diversity among five GI.1–RHDVs (one GI.1–RHDV HA+, three HA− and one HA+/− viruses) and five GI.1–RHDVa viruses (three HA+ viruses and two HA− viruses), which were assessed between 0 and 48 h after infection of the rabbits, based on nine indices of non-specific cellular immunity and three of non-specific humoral immunity, showed mainly decreases in their values in terms of indices of non-specific cellular immunity, falling at 4–8 h and 24–36 h after the infection of the rabbits. The highest number of changes, in the form of decreases, was registered with HA+ viruses, mainly with GI.1a–RHDVa viruses, while the lowest number of changes was also registered with GI.1–RHDVa but HA− viruses, indicating that the changes registered are related to their ability to form an antigenic variant (currently the GI.1a–RHDVa virus) and haemagglutination. These results [96], and analysis concerning the 10 viruses studied, allowed three immunotypes to be distinguished: I—the most immunogenic formed by 5 viruses, including 2 GI.1–RHDV HA− viruses, 1 HA+/− virus, 1 GI.1a–RHDV HA+ virus and 1 HA− virus; II—a moderately immunogenic one consisting of 3 viruses, namely 1 GI.1–RHDV HA+, 1 HA− virus and 1 GI.1a–RHDVa HA+ virus; and III—weakly immunogenic consisting of 1 GI.1a–RHDVa HA+ virus and 1 RHDVa HA− virus. The analysis [96] concerning the affiliation of the tested viruses to these three immunotypes does not fully indicate that their affiliation to the immunotypes is determined by their ability to haemagglutinate and form an antigenic variant. It is because the immunotype, mainly I, is poorly represented by HA+ viruses and antigenic variants of RHDV–RHDVa (currently GI.1a–RHDVa), which have been shown in studies (Section 2.1 and Section 2.6) to be associated with pathogenicity and immunogenicity. Also, the observations of Hukowska-Szematowicz [60] concerning the immunogenicity assessment of 13 GI.1–RHDV HA+ and 3 GI.1a–RHDVa HA+ viruses, conducted between 0 and 72 h after the infection of rabbits in terms of nine indices of non-specific cellular immunity, three parameters of non-specific humoral immunity, four indices of specific cellular immunity and two parameters of specific humoral immunity, showed their immunological differentiation. Changes in the immune pattern in these rabbits [60] were characterised by both increases and decreases in the indices studied, with the greatest number occurring in the order of non-specific cellular immunity, specific cellular immunity and non-specific humoral immunity and specific humoral immunity, and which occurred 4–8 h and 24–36 h after infection and were determined as in previous observations [101] by their ability to haemagglutinate and form antigenic variants. This study [60] also showed that there were three immunotypes among the 16 viruses assessed: I—the most immunogenic (2 GI.1–RHDV HA+ viruses); II—the moderately immunogenic (8 GI.1–RHDV HA+ viruses and 2 GI.1a–RHDVa HA+ viruses); and III—the least immunogenic (3 GI.1–RHDV HA+ viruses and 1 GI.1–RHDVa HA+ virus), which confirmed that they are associated with the ability to haemagglutinate and with the formation of an antigenic variant by these viruses, and which are related to the pathogenicity and immunogenicity of the viruses studied (Section 2.1 and Section 2.6). Also, in a study by Działo [15] conducted on five GI.1–RHDV viruses (three HA+ viruses and two HA− viruses), which were assessed at 0–60 h after infection in rabbits, analysing nine indicators from non-specific cellular immunity, three non-specific humoral immunity, four specific cellular immunity and two specific humoral immunity, showed their immunological diversity. The changes found in these studies [15] were characterised as in previous observations [60] by both an increase and a decrease in the parameters studied, falling 8–60 h after infection of the rabbits, except that they were most pronounced in the order of non-specific cellular immunity, specific cellular immunity, non-specific humoral immunity and specific humoral immunity and were related to the haemagglutination capacity of these viruses. Based on these results, the viruses evaluated were divided into three immunotypes: I—the most immunogenic, formed by one GI.1–RHDV HA+ virus; II—the moderately immunogenic—two GI.1–RHDV HA+ viruses and one GI.1–RHDV HA− virus; and III—the least immunogenic formed by one GI.1–RHDV HA− virus, which indicate a close relationship with the haemagglutination capacity of these viruses—a trait related to their pathogenicity and immunogenicity (Section 2.1). In contrast, the study by Niedźwiedzka-Rystwej et al. [117] was also conducted on five viruses defined as antigenic variants of RHDV–RHDVa (now GI.1a–RHDVa virus), among which three viruses were HA+ and two HA−, and which were assessed between 0 and 36 h after the infection of rabbits, in terms of four indicators of specific cellular immunity and one of specific humoral immunity, also showed their different immunogenicity, based on which only two immunotypes were determined: I—more immunogenic, represented by two GI.1a–RHDVa HA+ viruses and one HA− virus; and II—less immunogenic, formed by two GI.1a–RHDVa HA− viruses. These results indicate that the resulting immunotypes among the five antigenic variants of RHDV studied—currently GI.1a–RHDVa—are partly related to their ability to form an antigenic variant and to haemagglutination, both of which are related to their pathogenicity and immunogenicity (Section 2.1 and Section 2.6). These observations [117] were confirmed in studies on six GI.1–RHDV HA+ viruses and one GI.1a–RHDVa HA+ virus, between 0 and 48 h after infection in rabbits, on nine indicators of non-specific cellular immunity and three of non-specific humoral immunity [115]. It has been shown in [115] that the changes were mainly in the indices of non-specific cellular immunity, fell between 8 and 24 h after infection of the animals, manifested mainly by an increase in the values of the indices studied and were most pronounced with six GI.1–RHDV HA+ viruses and less so with one GI.1a–RHDVa HA+ virus. These studies [115] distinguished among these seven viruses assessed as in previous observations [117], only two immunotypes: I—the more immunogenic one (three GI.1–RHDV HA+ viruses); and II—the less immunogenic one (three GI.1–RHDV HA+ viruses and one GI.1a–RHDVa HA+ virus), which would indicate that the evaluated characteristics (pathogenicity, immunogenicity) of the tested viruses are mainly related to their haemagglutination capacity and only partly to their formation of antigenic variants (currently GI.1a–RHDVa). The results of these studies [115,117], and concerning immune differentiation, i.e., immunotype formation, are confirmed by observations carried out on six GI.1–RHDV HA+ viruses and one GI.1a–RHDVa HA+ virus, between 0 and 48 h after infection in rabbits, assessed for four indicators of specific cellular immunity and one for non-specific humoral immunity [116]. These latter results [116] are also similar in terms of immunological diversity to the results of a study on three GI.1–RHDV HA− viruses, which were assessed between 0 and 36 h after infection in rabbits, in terms of four parameters of specific cellular immunity and one parameter of specific humoral immunity [91].

To summarise the results of studies [15,33,60,91,96,115,116,117] concerning the relationship between the immunotypes, and pathogenicity and immunogenicity, of 33 GI.1–RHDV viruses and 15 GI.1a–RHDVa viruses, previously identified as antigenic variants of RHDVa (now GI.1a–RHDVa) characterised by different haemagglutination capacities, it should be stated that this relationship among the viruses studied was registered. This picture, in terms of the main immunogenicity of GI.1a–RHDVa viruses, is confirmed by studies [114] concerning their evaluation in terms of inducing indicators of natural and acquired immunity in rabbits infected with them. It should be added that the results of the studies presented [15,33,60,91,96,115,116,117] concerning the prevalence of immunotypes among the GI.1–RHDV and GI.1a–RHDVa viruses studied are mainly determined by indicators of non-specific and specific cellular immunity, which are related to the ability of these viruses to haemagglutinate and form an antigenic variant, features that influence their pathogenicity and immunogenicity (Section 2.1, Section 2.6 and [15,33,60,91,96,115,116,117]).

### 2.4. Lagovirus europaeus GI–RHDV Haematotypes and Their Pathogenicity (Infectivity) and Immunogenicity

The demonstrated relationship between phylogenogroups and immunotypes of *Lagovirus europaeus* GI–RHDV viruses and their pathogenicity (infectivity) and immunogenicity, for the haematotypes of these viruses, is recorded against the GI.1–RHDV and GI.1a–RHDVa viruses (Figure 2) [15,33,97,101,102] (such studies are lacking against the GI.2–RHDV2/b and RCV–GI.3 and GI.4 viruses). By investigating this relationship, which was determined at 0–60 h after the infection of rabbits, against 10 GI.1–RHDV viruses (8 HA+, 1 HA−, 1 HA+/−) [33], it was shown that, in most of the nine haematological blood indices assessed, a decrease was found more often than an increase in their values. The decrease concerned the number of leukocytes and lymphocytes (4–60 h) in rabbits infected with four GI.1–RHDV HA+ viruses, one GI.1–RHDV HA− virus and one GI.1–RHDV HA+/− virus, and the haemoglobin concentration (24–60 h) in rabbits infected with three GI.1–RHDV HA+ viruses, while the increase concerned only the number of neutrophils (4–60 h) in rabbits infected with six GI.1–RHDV HA+ viruses and one GI.1–RHDV HA+/− virus, indicating that the recorded changes in the blood picture of rabbits are related to the ability of the tested viruses to haemagglutinate—a feature affecting the pathogenicity and immunogenicity of these viruses (Section 2.1 and Section 2.3). The changes obtained among the 10 viruses tested resulted in the distinction of three haematotypes: I—with the greatest number of lesions induced (1 GI.1–RHDV HA+ virus); II—causing fewer lesions (2 GI.1–RHDV HA+ viruses); and III—giving the least number of lesions (5 GI.1–RHDV HA+ viruses, 1 HA− virus and 1 HA+/− virus), indicating that the haematotypes obtained are not unequivocally linked to the haemagglutination capacity of the viruses evaluated, because, among others, most of the HA+ viruses studied form haematotype III. Other studies [101], also concerning analogous parameters to previous studies [33] in five GI.1–RHDV viruses (one HA+, one HA+/− and three HA− viruses) and five GI.1a–RHDVa (three HA+ and two HA− viruses), conducted between 0 and 60 h after the infection of rabbits, showed a similar pattern of changes as in the above studies [33]. In these studies [101], the greatest changes were registered between 4 and 8 h and 24 and 36 h after the infection of rabbits, in terms of a decrease in platelet counts and blood protein counts, except that, in the first case, these affected rabbits were infected with only one GI.1a–RHDVa HA−, one GI.1–RHDV HA+ and two GI.1–RHDV HA− viruses, while in the second case, rabbits were infected with one GI.1a–RHDVa HA− virus. The image of these lesions [101] made it possible to distinguish among the nine viruses studied. Only haematotype I caused changes in the haematological picture in rabbits infected with one GI.1a–RHDVa HA− and three GI.1–RHDV viruses, including one HA+, one HA− and one HA+/−, and haematotype II caused slightly fewer changes in two GI.1–RHDV HA− viruses and four GI.1a–RHDVa virus, including three HA+ and one HA−. This affiliation of the viruses studied to haematotypes I and II indicates a relatively weak relationship between their haematotype formation and their ability to haemagglutinate and form the antigenic variant of the RHDV–RHDVa virus, now GI.1a–RHDVa virus. Similar observations to the above [101] were also obtained by studying four GI.1–RHDV HA+ viruses [102], assessed at 0–72 h post-infection in rabbits, in terms of analogous haematological indices as in previous studies [33,101]. In contrast, studies [15] conducted on five GI.1–RHDVs, including three HA+ and two HA−, assessed between 0 and 60 h after the infection of rabbits for analogous haematological indices as in previous studies [33,101,102], showed mainly a decrease in the values of the indicators studied in terms of platelet count (12–60 h) in rabbits infected with three GI.1–RHDV HA+ viruses and one HA− virus, and leukocyte count (8–56 h) in rabbits infected with three GI.1–RHDV HA+ viruses and one HA− virus, indicating that the changes observed are related to the ability of the viruses studied to haemagglutinate. Furthermore, the pattern of changes obtained in these studies [15] allowed three haematotypes to be distinguished among these viruses: I inducing the greatest number of lesions (one GI.1–RHDV HA+); II inducing a medium number of lesions (two GI.1–RHDV HA+); and III inducing a small number of lesions (two GI.1–RHDV HA−), confirming the results obtained with these viruses, that the haematotypes created by these viruses are related to their ability to haemagglutinate—a feature affecting their pathogenicity and immunogenicity (Section 2.1 and Section 2.3). This picture of change [15] was not confirmed in observations carried out on three GI.1–RHDV viruses, including two HA− and one HA+/−, assessed at 0–36 h after the infection of rabbits [97], in terms of the same blood parameters as in previous studies [15,33,101,102]. In these studies [97], only a variation was found in the haematological pattern of the viruses evaluated, manifested by an increase as well as a decrease in the blood indices studied, between 4 and 36 h after the infection of rabbits, which could not be linked to the haemagglutination capacity of these viruses, nor did these changes allow haematotypes to be distinguished among them.

To summarise studies on 32 *Lagovirus europaeus* GI–RHDV viruses, including 27 GI.1–RHDV viruses (14 HA+, 10 HA− and 3 HA+/−) and 5 GI.1a–RHDVa viruses (3 HA+ and 2 HA−) [15,33,97,101,102], it should be concluded that the picture obtained indicates their haematological differentiation but not constant conditioning of the formation of haematotypes by these viruses, which, to a lesser extent than with phylogenogroups and immunotypes, are related to their haemagglutination capacity and the formation of the antigenic variant RHDV–RHDVa (now GI.1a–RHDVa), features that are related to and influence the pathogenicity and immunogenicity of the viruses evaluated [8,24,26,33,47,56,60,85,91,92,96,97,98,100,101,102,107,118,119]. In addition, among these studies are observations [60,97,100,101,102] in which it was shown that GI.1a–RHDVa viruses, previously referred to as RHDV–RHDVa antigenic variants (now GI.1a–RHDVa) relative to GI.1–RHDV viruses, cause more remarkable haematological changes, which was also confirmed in the lesion pattern of rabbits infected with GI.2–RHDV2/b viruses, previously referred to as RHDV–RHDVb or RHDV2 variants (now GI.2–RHDV2/b) [18,83,120] and which documents the interaction of the antigenic variant and RHDV variant on their haematological differentiation. These recent studies [18,83,120] showed that GI.2–RHDV2/b viruses in infected rabbits cause a decrease in white blood cell counts and platelet counts, which are mainly recorded a few hours before the death of the test animals, which would further indicate that these changes in rabbits are related to their pathogenicity—a feature that interacts with their immunogenicity [55,121]. The cited studies on GI.1a–RHDVa viruses [60,97,100,101,102] and GI.2–RHDV2/b viruses [18,55,83,120,121] indicating a link between the haematological changes they cause and their pathogenicity and immunogenicity were also confirmed in studies [15,33,101,102], which indicated that in rabbits infected with GI.1a–RHDVa viruses, previously identified as the antigenic variant of RHDV–RHDVa, there is an association between the changes in the haematological picture, the formation of haematotypes by these viruses and their pathogenicity, which is linked to their immunogenicity [33,55,60,85,97,100,101,102,121].

### 2.5. Lagovirus europaeus GI–RHDV Virus Pathotypes and Their Pathogenicity (Infectivity) and Immunogenicity

When assessing the existence of a relationship between the pathotypes of these viruses (Figure 2) and their pathogenicity and immunogenicity, it should be noted that by studying the pathogenicity of 10 GI.1–RHDV viruses with different haemagglutination capacities (8 HA+, 1 HA+/− and 1 HA−) with which rabbits were infected, they were found to form three pathotypes [33]. Pathotype I, causing a mortality of 80–100%, was formed by six HA+ viruses and one HA+/− virus; pathotype II, giving a mortality of 60–80%, was formed by one HA+ and one HA− virus; and the pathotype III, causing a mortality of less than 60%, was formed by one HA+ virus, indicating that the pathotypes of these viruses are related to their haemagglutination capacity, a feature that determines their pathogenicity and thus their immunogenicity [10,85,91,96,97,98,114]. In other studies [15] conducted on five GI.1–RHDV viruses also differentiated by their ability to haemagglutinate (three HA+ and two HA−), it was shown that they comprise only two pathotypes, with I causing 100% mortality (three HA+ strains) and II causing a mortality rate of 62.5 to 87.5% (two HA− strains), which would confirm the results of previous studies [33]. The results of these two studies [15,33] are also consistent with the results of observations by Hukowska–Szematowicz [60], in which 16 GI.1–RHDV HA+ viruses were evaluated, including 3 GI.1a–RHDV HA+ viruses (formerly the antigenic variant of RHDV–RHDVa), among which two pathotypes were also identified, except that I caused a mortality rate of 60–100% (12 GI.1 HA+ viruses and 3 GI.1a HA+ viruses), while II caused a mortality rate of less than 60% (1 GI.1–RHDV HA+ virus). These results [60] demonstrate that the isolated pathotypes of the viruses studied are linked not only to their ability to haemagglutinate but also to the formation of antigenic variants of RHDV–RHDVa (now GI.1a–RHDVa virus), which are more immunogenic and pathogenic [114], which would confirm the results of other studies ([10,85,91,96,97,98] and Section 2.1 and Section 2.6), in which the pathotypes of these viruses are associated with their pathogenicity and immunogenicity. In contrast, studies [100] conducted on five GI.1–RHDV viruses with a different haemagglutination capacity (one HA+, one HA+/−, three HA− viruses) and five GI.1a–RHDVa viruses (formerly RHDV–RHDVa antigenic variant), also with a different HA capacity (four HA+ viruses and one HA− virus), distinguished two pathotypes among them. Type I caused a mortality rate of 90–100% (one GI.1–RHDV HA+ virus, one HA+/− virus, three HA− viruses and three GI.1a–RHDV HA+ viruses, one HA− virus), and II caused a mortality rate of 30% (one GI.1a–RHDVa HA+ virus), and these studies also indicate a link between their pathotype formation depending on their formation of the RHDV–RHDVa antigenic variant (now GI.1a–RHDVa) and their haemagglutination capacity.

To summarise the results of the studies presented [15,33,60,100], as well as the results of other authors ([10,85,91,96,97,98,114] and Section 2.1 and Section 2.2), concerning the association of the ability of GI.1–RHDV (formerly RHDV) and GI.1a–RHDVa (formerly RHDVa) between their ability to haemagglutinate and form the antigenic variant RHDV–RHDVa and the antigenic variant RHDV–RHDVb or RHDV2, and their pathogenicity and immunogenicity, it should be stated that such a relationship registers. Observations concerning this relationship, both in terms of the association of immunogenicity and pathogenicity with the formation of an antigenic variant by these viruses, i.e., virus GI.1–RHDVa, were also confirmed in studies in rabbits infected with the RHDV variant, now GI.2–RHDV2/b [39], which is assumed to be due to its greater immunogenicity [114], increasing immunity in susceptible animals, which reduces their mortality, and which in practice results in its more widespread occurrence in the wild, relative to GI.1–RHDV viruses, including GI.1a–RHDVa. This greater immunogenicity of these viruses is associated, in rabbits infected with this virus, with changes in MHC class II and I genes, CD4+ and CD8+ T lymphocyte and T cell activity, NK cell, APC and PMN activity and IFN-γ activity, as well as in unspecified indices of innate immunity associated with physiological and immune factors [19,38,79,121,122,123].

### 2.6. Change and Expansion of the Infectious Spectrum Resulting from the Creation of Antigenic Variant and Lagovirus europaeus GI–RHDV and Their Pathogenicity (Infectivity) and Immunogenicity

**Figure 3:**
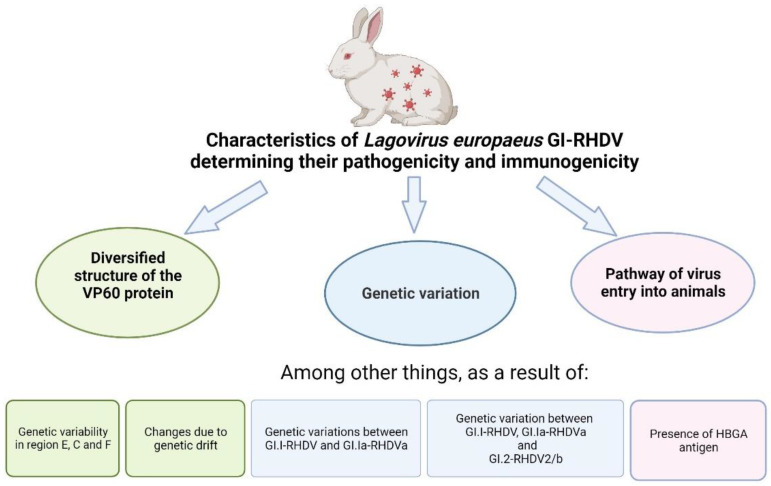
Characteristics of *Lagovirus europaeus* GI-RHDV that determine their pathogenicity and immunogenicity and influence their infectious spectrum. This figure was drawn with BioRender.com Descriptions: see main text for details.

The change and expansion of the infectious spectrum of *Lagovirus europaeus* GI–RHDV viruses, associated with the formation of RHDV (now GI.1–RHDV virus), the RHDV–RHDVa antigenic variant (now referred to as GI.1a–RHDVa), and the RHDV–RHDVb or RHDV2 variant (now referred to as GI.2–RHDV2/b), is related to their pathogenicity and immunogenicity. It is the characteristics of these viruses that have caused GI.1a–RHDVa (an antigenic variant of RHDV) to exhibit pathogenicity not only for domestic (*O. cuniculus* domesticus) and wild (*O. cuniculus*) rabbits and GI.2–RHDV2/b (variant RHDV) for domestic rabbits (*O. cuniculus* domesticus), including young rabbits up to 4 weeks of age and immunised against the disease caused by this virus, but also, in addition to wild rabbits—*O. cuniculis*, four other wild rabbit species (*S. auduboni*, *S. nuttali*, *S. floridanus*, *Romegolagus diazi*) and seven different species of hares (*Lepus* (*L.*) *timidus*, *L. timidus hibernicus*, *L. europaeus*, *L. capensis* subsp. Mediterraneus, *L. corsicanus*, *L. californicus*, *L. alleni*), as well as wild ruminants—mountain muskrat (*Moschus sifanicus*) and *European badger* (*Meles meles*) [4,6,9,10,13,14,16,18,19,26,40,41,42,43,44,45,46,47,48,49,50,51,52,53,54,55,56,57]. It should be reported that, as mentioned, the original RHD virus (now GI.1–RHDV) was reported in 1984 in China, as the cause of highly pathogenic disease in domestic rabbits (*O. cuniculus* domesticus) and subsequently wild rabbits (*O. cuniculus*) [36], which in subsequent years was recorded in many areas of the world [4,10,11,15,18,21,33,41,54,59,60,61,62,63,64,65,66]. RHD virus (now GI.1–RHDV), including GI.1a–RHDVa, the antigenic variant of RHD virus, was assumed to cause symptomatic infections only in adult rabbits, while in rabbits over 4–5 weeks of age, it was initially assumed to cause asymptomatic infections. However, it was later shown to cause symptomatic infections in these rabbits [10,124,125]. After 2010, these infections have been recorded not only in domestic rabbits, including young rabbits up to 4 weeks of age (even 11 to 15 days old) and rabbit haemorrhagic disease immunised rabbits, but also in the five wild rabbit species and seven hare species mentioned above, and in wild ruminants and the *European badger* [4,6,9,10,13,14,16,18,19,26,40,41,42,43,44,45,46,47,48,49,50,51,52,53,54,55,56,57], infections which were caused by GI.2–RHDV2/b viruses, i.e., a variant of RHDV. These facts indicate that the original RHD virus (now GI.1–RHDV) has altered and broadened its infectious spectrum and broken the genus (rabbit, including wild rabbits, hare, ruminants and badger) and species (different species of wild rabbits and hares) barriers, and this is associated with a change in their pathogenicity and immune reactivity (immunogenicity and antigenicity of these viruses), [6,8,9,10,11,13,18,45,56,64,72,82,109,126,127] resulting from their different conformation within the VP60 protein, their different genetic conformation and their route of entry into susceptible animals (rabbits, hares) (Figure 3).

Discussing the first feature of these viruses (Figure 3) that influences their pathogenicity and immunogenicity, that is, the variable structure within their VP60 protein, it should be stated that it influences the variation and expansion of their infectious spectrum since it has been shown that among the genes of the VP60 protein of GI.1–RHDV (now GI.1–RHDV), the most variable region, among the variables, is the E region, and among the others, the C and F regions; hence, it is assumed that the variation in these regions in these viruses contributes to creating the conditions for the occurrence within them of the antigenic variant RHDV–RHDVa (now GI.1a–RHDVa) and the variant RHD–RHDVb or RHDV2 (now GI.2–RHDV2/b) [8,9,13,33,45,53,64,102,126,127]. This thesis is supported by the study of Qi et al. [79], in which it was shown that variation resulting from an amino acid change in the sequences encoding the VP60 protein of this RHD virus (now GI.1–RHDV) is a sufficient condition for the ‘emergence’ of the antigenic variant RHDV–RHDVa (now GI.1a–RHDVa virus) and the variant RHDV–RHDVb or RHDV2 (now GI.2–RHDV2/b virus). In addition, these studies [79] indicated that the relationship and dependence of the conformation of GI.1a–RHDVa virus and GI.2–RHDV2/b virus on their pathogenicity is probably also influenced by the N-glycosylation of their VP60 protein, while on their pathogenicity and immunogenicity, the phosphorylation of this protein. When discussing the conditions leading to changes in the pathogenicity and immunogenicity of these viruses, in the context of their formation of the RHDV–RHDVa antigenic variant, it should be stated that the term RHDV–RHDVa antigenic variant [8,9], now GI.1a–RHDVa virus [8,9], was introduced based on its antigenicity, i.e., only specific antibody binding to epitopes of the VP60 viral protein. However, no consideration was given to its immunogenicity, i.e., the ability to elicit a specific response against itself, as has now been demonstrated [96,100], which would confirm that the structure of this virus is also related to its immunogenicity. It should be added that the first described antigenic variants of RHDV–RHDVa (now GI.1a–RHDVa) [1] were the Italian antigenic variant Pv97 and Vt97 [9] and the German variants Tripitis and Hartsmanndorf [90], of which more than 100 have been registered in many parts of the world [10], including Poland [64,88], and which, relative to GI.1–RHDV, are characterised by greater pathogenicity and immunogenicity [4,8,9,15,33,53,56,60,64,90,96,99,100,107,114]. Also, the RHDV variant RHDVb or RHDV2, described for the first time in France [13] (GI.2–RHDV2/b [1]), currently registered at around 300 [88] in various regions of the world [10,26,38,67,128], including in Poland [129], is now more widely represented in the environment than GI.1–RHDV, including the GI.1a–RHDVa virus [6,17,18,24,37,41,43,47,53,56,72,84,118,119,130,131,132,133]. However, in the literature, apart from two papers [55,121], there are no studies assessing this virus’s immune reactivity. An analogous situation to the now more common occurrence of GI.2–RHDV2/b viruses in the environment was also recorded at the time of the emergence of the GI.1a–RHDVa virus, which also displaced GI.1–RHDV viruses from the environment [4,10], and which, together with RCV–A1 viruses in wild rabbits, are currently being displaced by GI.2–RHDV2/b viruses [4,6,13,17,18,24,26,37,41,43,47,56,72,84,118,119,130,131,132,133] and their recombinants [11,12,21,47,54,72,82,83,84,134]. Hence, it can be assumed that the appearance in the environment of the antigenic variant of RHDV–RHDVa (now GI.1a–RHDVa virus) and the variant of RHDV–RHDVb or RHDV2 (now GI.2–RHDV2/b) is the result of their variability, as a result of the evolution of the original virus termed RHD (now GI.1–RHDV), in terms of their change in pathogenicity and immunogenicity [4,11,12,54,72,84]. This change has resulted in the GI.1a–RHDVa virus (formerly antigenic variant RHDV–RHDVa) being pathogenic to domestic rabbits (*O. cuniculus* domesticus) and wild rabbits (*O. cuniculus*), while the GI.2–RHDV2/b virus (formerly the RHDV–RHDVb or RHDV2 variant) is pathogenic not only against domestic rabbits, including young rabbits under 4 weeks of age and rabbits immunised against rabbit haemorrhagic disease, but also four additional species of wild rabbits and seven species of hares, as well as wild ruminants: the mountain muskrat and European borax [4,6,9,10,13,14,16,18,19,26,40,41,42,43,44,45,46,47,48,49,50,51,52,53,54,55,56,57]. It is indicated that these changes in pathogenicity and immunogenicity of the characterised viruses, which are part of their change in the infectious spectrum, are due to changes resulting from genetic drift in these animals [4,11,12,135,136], including as a result of selection for differentiation of their genes for MHC antigens [32,66,135]. It has also been suggested that variation in these viruses, mainly in immunogenicity, is also associated in rabbits with the HBGA, SEC1 and FUT2 genes [137,138], PINK1, PARKIN, FUNDC1, BNIP3, BNIP3L, VDAC and MFN1 genes [99] and genes included in xylosyl- and glucuronyltransferases, namely LARGE2 and EXTL1 [66], as well as genes related to the metabolic pathway of the Golgi apparatus and genes conditioning inflammation [103], elements that play an important role in the development of immune responses in rabbits after infection with these viruses, and which determines the incidence of the disease caused by them in these animals.

A second feature (Figure 3) related to the pathogenicity and immunogenicity of the antigenic variant RHDV–RHDVa (now GI.1a–RHDVa) and the RHDV–RHDVb or RHDV2 variant (now GI.2–RHDV2/b), which alter and expand their infectious spectrum, is the difference in their genetic structure [6,8,9,10,12,13,37,45,47,53,56,60,72,81,82,83,108,109,139]. The variation in the genetic structure of GI.1–RHDV and GI.1a–RHDVa viruses is 7% [9], and between GI.1–RHDV viruses, including GI.1a–RHDVa, and GI.2–RHDV2/b, at the 7–9% level [9,13,56,81,139], including at the nucleotide level 14–16–21% [18,130,134] and the amino acid level 8–18% [72], although the nucleotide homology of GI.1a–RHDVa viruses is determined to be 96–99% and of GI.2–RHDV2/b viruses to be 98–100% [18]. It was also recorded [13,16,43,81] that the difference in the genome sequence of pathogenic GI.2–RHDV2/b viruses, relative to non-pathogenic and low pathogenic *Lagovirus europaeus* GI–RHDV viruses, was less than that of pathogenic GI.1–RHDV, including GI.1a–RHDVa, because it has been shown that the dissimilarity in nucleotide structure between GI.2–RHDV2/b viruses and non-pathogenic RCV viruses was 27–30%, while between GI.2–RHDV2/b and GI.1a–RHDVa viruses it was 24–28% [13,16,43,45,64,81,140]. However, it is indicated that the latter two viruses are highly related [79]. In contrast, this difference in nucleotide structure between the variant RHDV virus (now GI.2–RHDV2/b) and the antigenic variant RHDV (now GI.1a–RHDVa) relative to the parental form of RHD virus (now GI.1–RHDV) in the structural genes is 3.9%, and in the non-structural genes 21.9% to 25%, although these data, and concerning their NSPs (non-structural proteins), are debatable, as it is also indicated that their genes may also originate from other RHDV genotypes, including the genotype of the non-pathogenic viruses GI.3 and GI.4–RCV [45,47,72,82,83,134]. The facts presented, relating to the variation in the genetic structure of the characterised viruses and their variation mentioned above in the structure of the VP60 protein, give rise to the conclusion that such a difference in the structure of these viruses (GI.1a–RHDVa and GI.2–RHDV2/b), leads to a differentiation in their pathogenicity (infectivity) and immunogenicity, and thus their change in pathogenicity, and this becomes the reason for the change and expansion of their infectious spectrum. This relationship in infectivity and their immunogenicity is also confirmed by observations from the immune response in young rabbits infected with the GI.1–RHDV and GI.2–RHDV2/b viruses [38], in which it has been shown that this picture is conditioned by and related to the expression of genes of NK cells, macrophages and cholangiocytes, as well as certain pro-inflammatory cytokines. In addition, it is indicated [38] that the immune status of young rabbits at about 4 weeks of age infected with GI.2–RHDV2/b is not only related, as previously assumed, to the interaction of specific antibodies obtained by young rabbits with colostrum from rabbits [124,125,128,141,142], but also with unknown immune factors [37,123], including possibly natural antibodies [19,38,123,141]. It should be added that the variation in the pathogenicity and immunogenicity of these viruses, and related to their different VP60 protein structure and their genetic make-up, which determine their formation of the antigenic variant RHDV–RHDVa, now GI.1a virus, and the variant RHDV–RHDVb or RHDV2 (now GI.2–RHDV2/b virus), is confirmed by immunological studies in rabbits infected with them [143]. It has been shown [143] that due to the infection of rabbits with GI.1a–RHDVa viruses, changes in the amount and activity of TNF-α, IL-6 and IL-10, as well as in the phagocytic activity of PMN cells and the cytotoxicity of NK cells and CD8+ T lymphocytes, as well as the ability to present antigen, have been registered. Such an association of the alteration and expansion of the infectious spectrum of RHDV viruses (now GI.1–RHDV) through the creation of an antigenic variant (GI.1–RHDVa) and an RHDV variant (GI.2–RHDV2Vb) with their pathogenicity and immunogenicity, has also been confirmed in clinical observations [19]. This study showed that the 84% mortality rate in rabbits due to GI.1–RHDV infection, compared to the 40% mortality rate in rabbits infected with GI.2–RHDV2/b, is related to their immunity, arising from the immunogenicity of these viruses. These results [19] have also been confirmed by several comprehensive immunological studies [15,19,33,38,60,66,79,85,91,96,98,99,100,101,102,114,115,116,117,121,122,144,145,146,147,148,149,150,151,152,153], in which the pathogenicity and immunogenicity of GI.1a–RHDV and GI.2–RHDV2/b viruses, the features that determine the alteration and expansion of their infectious spectrum, were shown to be related to multiple immune determinants in infected rabbits. It was shown in these observations that the immune status in these rabbits is mainly related to the activity of phagocytosis and apoptosis of PMN cells, leukocyte oxygen-dependent (MPO, ROS) and oxygen-independent (LZM) cidal capacity, the number of T cells, including TCD4+, TCD5+, TCD8+ and TCD25+, B cells and serum immunoglobulins, mainly IgG, but also the apoptosis and cytotoxicity of these cells, the expression of MHC class II and class I and the number of NK cells, APCs, cholangiocyte macrophages and Treg lymphocytes, as well as the amount (activity) of IFN-γ, IFN-α, TNF-α, IL-1β, IL-4, IL-6 and IL-10. This pattern of changes was confirmed in a study by Zhang et al. [154] in which it was shown that both natural and artificial infection of domestic rabbits with GI.1–RHDV results in changes in its presentation by MHC antigens to T lymphocytes, while infection with GI.2–RHDV2/b of wild-type rabbits results in an increased expression of MHC class I genes [32,103]. Furthermore, it has been shown that immunity in domestic rabbits infected with the RHDV–RHDVb or RHDV2 virus variant (now GI.2–RHDV2/b virus) is also conditioned by the effect of physiological [38] and pathological [19,143,155] bacterial intestinal flora of these animals. It has been recorded [156] that the stimulation of the gastrointestinal immune system in rabbits by microorganisms, that is, at the point of entry of the viruses in question into the macroorganism of susceptible animals, results in an increase in local intestinal immunity, conditioned mainly by SIgA and IgG. Hence, it can be assumed that the results regarding the immune status in rabbits infected with GI.1a–RHDVa and GI.2–RHDV2/b, i.e., viruses forming an antigenic variant and a variant of RHD virus, prove that they create fully the immune response of susceptible animals to these infections. This has also been confirmed in observations [32,55,103,130], in which it was shown that this picture is also linked to changes in the determinants of natural and acquired immunity in infected rabbits, including those resulting from changes in the expression of genes related to the process of autophagy and mitophagy—important phenomena in viral infections [99,105,157]. It has also been documented that the immunity of animals infected with viruses forming the antigenic variant RHDV–RHDVa (now GI.1–RHDV) and the variant RHDV–RHDVb or RHDV2 (now GI.2–RHDV2/b), which is related to their pathogenicity and immunogenicity—features that determine their alterations and extensions of the infectious spectrum, is also linked to natural antibodies [19,38,123,141] and probably defensins [158] and TLR3, TLR4 receptors [99] and RLR [159]. It has also been pointed out [153] that the immune status in rabbits infected with RHDV (now GI.1–RHDV) is also associated with the activity of IFN-γ resulting from the presence in these animals in their erythrocytes of β-haemoglobin, which, by promoting the expression of this cytokine, increases the activity of reactive oxygen species, which contributes to the inhibition of the replication of this virus and which leads to a reduction in its amount and therefore its virulence (virulence), a feature that determines its pathogenicity and immunogenicity.

A third feature (Figure 3) explaining the relationship between the pathogenicity and immunogenicity of the characterised viruses and their infectious spectrum, as a result of their formation of the antigenic variant RHDV–RHDVa (now GI.1–RHDV) and the variant RHDV–RHDVb or RHDV2 (now GI.2–RHDV2/b), is the pathway of entry of these viruses into rabbits, including young rabbits up to 4 weeks of age and hares. It is related to the HBGA (histo-blood group antigen—α(1,2)fucosylated glycans) antigens found on the surface of the cells of the gastrointestinal tract (mainly duodenum) and respiratory tract (mainly trachea) of these animals [109,137,139,160,161,162,163], although possibly also other receptors specific to these sites [4]. It should be added that the evidence for the involvement of HBGA antigens in the infection of rabbits with Lagovirus europaeus GI–RHDV is provided by the results of studies in which it was shown by blocking with antibodies 1B8, 5H3 for VLP–RHD HBGA antigens that the sites of their association with these viruses is their outermost capsomer fragment, i.e., the epitope of the P2 subdomain occurring at the 326–331 and 338–342 amino acid sites of the VP60 protein of these viruses [161]. Furthermore, it has been recorded that in small intestinal and respiratory epithelial cells in rabbits, including rabbits up to 4 weeks of age and hares, there is a wide variation in the expression of HBGA antigens as specific entry sites for these viruses [14,109,137,139,160,161,162,163]. GI.1–RHDV, including GI.1a–RHDVa, which infects adult and young rabbits but over 4–5 weeks of age and causes rabbit haemorrhagic disease, has also been shown to have a high affinity for HBGA antigens present in the gastrointestinal and respiratory tracts of these animals, while GI.2–RHDV2/b shows an analogous affinity to these antigens, not only in adult rabbits but also in young rabbits up to 4 weeks of age and hares, which explains the higher morbidity and incidence of GI.2–RHDV2/b virus infection in these animals [14,109,137,139,160,161,162,163].

Furthermore, an additional fact confirming the higher morbidity and incidence of adult rabbits, relative to young rabbits, of infection with these viruses concerning HBGA antigens is the higher expression in adult rabbits of the FUT1 gene, encoding an enzyme belonging to the α-1,2-fucosyltransferases necessary for the production of HBGA antigens [38]. In this study, a lower expression of this gene was shown [38] at 24 h after the infection of rabbits with GI.1–RHDV in relation to a higher expression of this gene at 12 h after infection of rabbits with GI.2–RHDV2/b, explaining the higher affinity of the latter virus towards young rabbits. The thesis of higher morbidity in rabbits, including young rabbits and hares after GI.2–RHDV2/b virus infection, due to HBGA antigens, is supported by studies showing a more intense presence and higher numbers of these antigens in the body of rabbits under 32 days of age, compared to 12-week-old rabbits [123] and the similar occurrence of these antigens in hares compared to rabbits [109,137,139,160,161,162,163]. According to Calvete et al. [19], the facts related to the higher frequency of HBGA antigens in rabbits’ young up to 4 weeks of age and hares fully document and explain the morbidity and incidence of GI.2–RHDV2/b virus infections in these animals. Furthermore, it has been shown by investigating the morbidity of mainly young rabbits to GI.2–RHDV2/b virus infection, in the context of the intensity of HBGA antigens found in these animals, that there is fucosylation of the cellular ligands for HBGA antigens, which further enhances the activation of these responses [38].

Summarising the data presented on the link between the pathogenicity (infectivity) and immunogenicity of *Lagovirus europaeus* GI.1–RHDV viruses resulting from and related to the different structure of their VP60 protein and their different genetic make-up, as well as their route of entry into susceptible animals, and the infectivity spectrum resulting from the formation of the antigenic variant RHD–RHDVa (now GI.1–RHDVa virus) and the variant RHDV–RHDVb or RHDV2 (now GI.2–RHDV2/b virus), it should be concluded that such a relationship exists and is close. It should be added that the data on the different structures, including the genetic structure and route of entry into the macro-organism of infected animals (Figure 3) of the characterised viruses and the resulting different pathogenicity and immunogenicity, is reflected in the immune status in rabbits infected with these viruses, which documents that their characteristics of pathogenicity and immunogenicity are associated with their widening of the infectious spectrum manifested by the breaking down of genus barrier (rabbit–hare–mountain muskrat–*European badger*) and species (different rabbit and hare species) barriers. It should also be added that evidence of a change and widening of the infectious spectrum of these viruses due to a change in their genetic structure is provided by the infection of the *European badger* with an intergenotypic recombinant of GI.4–RHDV virus and GI.2–RHDV2/b virus [50], in which the structure of the recombinants as shown by [4,11,21,54,72,84], are ‘stabilised’ by the non-structural genes of non-pathogenic viruses and the structural genes of pathogenic viruses. However, this thesis is not confirmed by Mahar et al. [12], who indicate that mainly the non-structural genes of recombinant viruses are the critical determinants of their stabilisation.

## 3. Other Features and Phenomena of *Lagovirus europaeus* GI–RHDV Viruses in the Context of Their Pathogenicity (Infectivity) and Immunogenicity

When characterising the characteristics of these viruses in terms of their pathogenicity and immunogenicity, it should be stated that an essential fact that affects the knowledge of this relationship is the impossibility of growing these viruses in vitro [153]. Although a method of obtaining RHDV (now GI.1–RHDV) viruses in vitro by transfection of cell lines has been described, it still does not offer the possibility of obtaining them, although the viruses obtained by this route are capable of causing rabbit haemorrhagic disease [164]. It has also been reported that a mutant RHDV (mRHDV) obtained [165], ‘created’ after the insertion of two amino acids, Arg and Asp, at the 305 and 307 sites of the VP60 RHDV protein, respectively, replicates in vitro and exhibits pathogenic activity like RHDV (now GI.1–RHDV) and induces the production of specific antibodies in infected rabbits. In these studies [165], it was also recorded that the mRHDV virus protein, containing the Arg-Gly-Asp (arginine–glycine–aspartic acid) amino acid sequence, referred to as ‘RGD’ (Arg-Gly-Asp) [165], has a site recognised by more than 20 integrins responsible for GI.1–RHDV virus adhesion to host cells, which would indicate a possible new potential pathway for its entry into the macro-organism of susceptible animals [166]. In terms of the entry route of this virus into the organism, it has also been shown [165] that, in the case of RK13 cells, the specific ‘entry’ of it during infection into this culture is the integrin receptor α3β1. It has also been recorded [167] that a nucleolin (NCL) protein present in many mammalian cells also supports the entry of this virus into susceptible animals [167]. This process occurs through the fusion of the N-terminal domain of nucleolin (aa 285–318) with the P1 subdomain (aa 468–484) of the VP60 protein of RHD virus (now GI.1–RHDV virus). This pathway has been shown to [168] greatly enhance its penetration into the cell interior of susceptible animals and occurs by clathrin-dependent endocytosis, following the fusion of the clathrin light chain A (CLTA) with the NCL [168,169]. Furthermore, it has been shown that this process is also mediated by the amino acid structure, at Positions 304–314, of the P2 subdomain of the VP60 protein of this virus [77]. The above facts, it seems, are also essential elements that may be relevant in the future when considering the determinants of pathogenicity (infectivity) and immunogenicity of Lagovirus europaeus GI–RHDV viruses.

## 4. Summary and Conclusions

(a) The primary rabbit haemorrhagic disease-causing RHD virus is heterogeneous since pathogenic (GI.1–RHDV, GI.1a–RHDVa and GI.2–RHDV2/b) and non-pathogenic (RCV) viruses have been distinguished within it, and which, due to changes in pathogenicity (infectivity) and immunogenicity, have altered and expanded their infectious spectrum. The result is that GI.1–RHDV, including GI.1a–RHDVa, is pathogenic only for domestic (*O. cuniculus* domesticus) and wild (*O. cuniculus*) rabbits, while GI.2–RHDV2/b is pathogenic not only for the rabbits mentioned above but also for domestic rabbits up to 4 weeks of age and rabbits immunised against haemorrhagic disease and various species of wild rabbits because, in addition to *O. cuniculus*, it is pathogenic for *S. audubonii*, *S. nuttallii* and *S. floridanus*, *Romegolagus diazi* and various hare species (*L. timidus*, *L. timidus hibernicus*, *L. europeaus*, *L. capensis* subsp. Mediterraneus, *L. corsicanus*, *L. californicus* and *L. alleni*), as well as wild ruminants: the mountain muskrat (*Moschus sifanicus*) and *European badger* (*Meles meles*).
(b) It is stated that this change in the pathogenicity and immunogenicity of pathogenic viruses belonging to Lagovirus europaeus GI–RHDV, which is the reason for the change and extension of their infectious spectrum, from domestic rabbits, including juveniles up to 4 weeks of age and vaccinated against rabbit haemorrhagic fever to five species of wild rabbits and seven species of hares, as well as wild ruminants and the *European badger*, is related to their capacity for haemagglutination, the formation of phylogenogroups, immunotypes and, to a lesser extent, haematotypes and pathotypes, and it is conditioned by their formation of antigenic variants and variants.
(c) The immune status of rabbits infected with these pathogenic viruses has been shown to be associated, in the following order:
  (c_1_) The process of phagocytosis and apoptosis of PMN cells, leukocyte oxygen-dependent (MPO, ROS) and oxygen-independent (LZM) cidal capacity and the number of T CD4, T CD5, T CD8, T CD25, B lymphocytes and serum immunoglobulins, mainly of the IgG class.
  (c_2_) The process of leukocyte apoptosis and cytotoxicity, including NK cell and T-cell cytotoxicity, cholangiocyte and APC cell activity and the expression of MHC antigens, mainly class II.
  (c_3_) The process of autophagy and mitophagy and such cytokines as IFN-γ, INF-α, TNF-α, IL-1β, IL-4, IL-6, IL-10 and probably natural antibodies, defensins and TLR-3, 4 and RLR receptors.
(d) In view of the observed high variability of *Lagovirus europaeus* GI-RHDV, manifested by the extension of their infectious spectrum, as well as the high immunological reactivity caused by them, mainly in terms of natural immunity, it is pointed out that future studies on these viruses in terms of assessing their immunogenicity should be conducted mainly in the area of natural immunity, including the activity of PMN cells, NK cells and cholangiocytes.

## Data Availability

Not applicable.
